# Assessing the efficacy and safety of Sildenafil *vs*. Nifedipine in improving endometrial blood flow and thickness in women with recurrent first-trimester miscarriage

**DOI:** 10.25122/jml-2023-0068

**Published:** 2023-06

**Authors:** Saba Al-Thuwaynee

**Affiliations:** 1 Department of Obstetrics and Gynecology, College of Medicine, University of Al-Qadisiyah, Al Diwaniyah, Iraq

**Keywords:** Nifedipine, Sildenafil, recurrent first-trimester miscarriage, uterine blood flow, resistive index, pulsatility index

## Abstract

Endometrial thickness and uterine blood flow influence pregnancy continuation until term. Nifedipine, a type II calcium channel blocker, and Sildenafil, a type 5-specific phosphodiesterase inhibitor, have shown the potential to improve these factors. This study aims to compare the safety and efficacy of Nifedipine and Sildenafil in improving endometrial blood flow and thickness in Iraqi women with recurrent first-trimester miscarriages. Women with unexplained recurrent pregnancy loss in the first trimester (non-pregnant during the study) were randomly assigned to two groups. Transvaginal color Doppler ultrasound assessed uterine artery pulsatility, resistance indexes, and endometrial thickness during the second phase of the menstrual cycle (day 15 to day 25). The first group received oral Nifedipine (10 mg) twice daily, while the second group received oral Sildenafil citrate (20 mg) every 8 hours from day 5 to day 25. Baseline measurements showed no significant differences in pulsatility index between the groups (2.02±0.52 for Nifedipine, 2.03±0.49 for Sildenafil, p=0.927). Sildenafil treatment resulted in a more noticeable reduction in the pulsatility index. The resistive index had a significant difference in baseline readings (0.98±0.14 for Nifedipine, 1.06±0.14 for Sildenafil, p=0.033), with Sildenafil showing a more pronounced reduction. Post-treatment, Sildenafil demonstrated a greater improvement in endometrial thickness than Nifedipine (10.09±0.74 mm *vs*. 9.34±0.50 mm, respectively; p<0.001). Both medications were safe and effective in improving endometrial blood flow and thickness in women with recurrent pregnancy miscarriages, with Sildenafil showing greater efficacy.

## INTRODUCTION

Recurrent pregnancy loss is a significant reproductive challenge, and its definition varies across countries. In the United States, it is characterized by failed clinical pregnancies in two or more consecutive attempts, with documentation based on histopathology and/or ultrasound [1]. In the United Kingdom, the criteria specify that three or more early pregnancies are lost consecutively [2]. The underlying etiology remains unknown in approximately 50% of cases [3]. Recurrent pregnancy loss can be classified as primary if no previous live birth has occurred or secondary if a previous live birth has been achieved [4].

Unexplained recurrent miscarriage represents three or more miscarriages in healthy women without any known underlying pathology. However, research suggests that a subset of women with unexplained recurrent miscarriages may have underlying pathologies contributing to their condition. As a result, there are two categories of unexplained recurrent miscarriages: type I, which happens by chance in females without an obvious pathology and the prognosis of such type, is good, and type II, which is associated with an undetected pathology not routinely identified by clinical methods and tends to have a poorer prognosis [5].

The etiology can be attributed to genetic abnormalities such as aneuploidy [6]. Anatomical abnormalities such as arcuate uteri, didelphic uterus, bicornuate uterus, unicornuate uterus, and septate uterus are also associated with recurrent pregnancy loss [7,8]. Some endocrine abnormalities, such as thyroid disease and diabetes mellitus, are also reported to be associated with recurrent pregnancy loss [9,10]. Antiphospholipid antibody syndrome is reported in many women with recurrent pregnancy loss [11]. Some bad habits, such as smoking, alcoholism, and excess caffeine consumption, have also been linked to an increased incidence of recurrent pregnancy loss [12]. Inherited thrombophilias have been reported in women with recurrent pregnancy loss [13].

The prevalence rate of recurrent pregnancy loss is much lower than spontaneous miscarriage [14]. The prevalence rate of recurrent pregnancy occurring before the twenty-first week of pregnancy ranges from 0.8 to 1.4 %. Nevertheless, when considering biochemical evidence such as a positive pregnancy test or elevated serum beta-hCG levels, the prevalence rate can be as high as 2% to 3% [15].

A competent blastocyst and a receptive uterus interact during the highly coordinated implantation process. Natural reproductive ability in humans indicates that implantation failure accounts for about two-thirds of lost pregnancies and that the chance of conception every cycle is relatively modest (30%). Optimum uterine artery blood flow and endometrial thickness are essential for successful implantation [16].

Two important factors that may affect the continuation of pregnancy until term are endometrial thickness and uterine blood flow [17]. Enhancing uterine blood supply by relaxing the smooth muscles of the uterine arteries can increase endometrial blood flow, leading to improved endometrial thickness and receptivity. This is the rationale for using Nifedipine, a type II calcium channel blocker [17]. On the other hand, when it comes to endometrial blood flow, the uterine artery plays the principal role. It has been suggested that the use of Sildenafil, a type 5-specific phosphodiesterase inhibitor, may improve blood flow by augmenting the level of the vasodilator substance nitric oxide through the inhibition of cyclic guanosine monophosphate (cGMP) [18, 19].

This research aimed to compare the safety and efficacy of Nifedipine *versus* Sildenafil in enhancing endometrial blood flow and thickness in Iraqi females with recurrent first-trimester miscarriages.

## MATERIAL AND METHODS

### Study design and participants

A randomized controlled clinical trial was conducted at the Maternity and Child Teaching Hospital in Adiwaniyah province, Iraq. The study was carried out from June 2021 through June 2022. A total of 60 women with a history of unexplained recurrent pregnancy loss in the first trimester, who were not pregnant at the time of the study, were randomly allocated into two groups.

The inclusion criteria included: age between 20 and 35 years, BMI<30 kg/m2, two or more recurrent first-trimester miscarriages, and regular menstrual cycle. The exclusion criteria included women with contraindications to either drug being administered, women with known pathological conditions that could cause miscarriage, obese women, and women with irregular menstrual cycles.

### Assessment and intervention

The participants underwent a comprehensive assessment during the second phase of the menstrual cycle (day 15 to day 25). This involved collecting their medical history and performing transvaginal color Doppler ultrasound to evaluate the pulsatility and resistance indexes of the uterine artery, as well as the endometrial thickness. The first group received Nifedipine (10 mg) by oral route twice daily, and the second group received Sildenafil citrate (20 mg) orally every 8 hours from day 5 of the menstrual cycle until day 25. Reassessment of pulsatility and resistance indexes of uterine artery and endometrial thickness was done at the second phase of the next menstrual cycle (day 15 to day 25) using transvaginal color Doppler ultrasound to observe the effect of these pharmacological agents.

### Statistical analysis

Data were analyzed using the Statistical Package for Social Sciences (SPSS) (IBM, Chicago, USA, version 16). Descriptive statistics were used to summarize the data, with categorical variables expressed as percentages and numbers and quantitative data presented as mean, median, standard deviation, range, and interquartile range. Statistical comparisons were performed using Student's t-test for comparing means, the Mann-Whitney U test for comparing mean ranks, and the Chi-square test (with Yates correction when applicable) for comparing proportions. The significance level was set at p≤0.05.

## RESULTS

The demographic characteristics of the enrolled women are presented in Table 1. There was no significant variation in the average age between the Nifedipine group (27.33±4.25 years) and the Sildenafil group (28.27±3.66 years) (p=0.365). However, there was a significant difference in the mean body mass index (BMI) of the Nifedipine group (24.13±2.54 kg/m2) and that of the Sildenafil group (25.97±2.08 kg/m2) (p=0.003). There were no significant differences in previous miscarriages between the two study groups (p=1.000).

**Table 1:** Demographic characteristics of participants

Characteristic	Nifedipine n = 30	Sildenafil n = 30	p
**Age (years)** Mean ±SD Range	27.33±4.25 20 -35	28.27±3.66 23 -34	0.365 I NS
**BMI (kg/m^2^)** Mean ±SD Range	24.13±2.54 20 -27	25.97±2.08 22 -29	0.003 I **
**Previous miscarriage** Median (IQR) Range	3 (1) 3 (4)	3 (1) 3 (4)	1.000 M NS

n: number of cases, SD: standard deviation, IQR: inter-quartile range, I: independent samples t-test, M: Mann Whitney U test, NS: not significant, **: significant at p ≤ 0.01

The mean HbA1c% and mean hormonal concentrations of Nifedipine and Sildenafil groups are presented in Table 2. There were no significant variations in average HbA1c% between the two groups, 5.58±0.69 % *versus* 5.41±0.55 %, respectively (p=0.267). There was also no significant variation in serum TSH between the study groups, 3.06±1.53 mIU/L *versus* 2.51±1.15 mIU/L, respectively (p=0.119). In addition, there was no significant variation in serum prolactin between study groups, 15.30±6.14 ng/ml *versus* 13.03±6.80 ng/ml, respectively (p=0.181).

**Table 2:** Comparison of HbA1c% and serum hormonal levels between Nifedipine and Sildenafil groups

Characteristic	Nifedipine n = 30	Sildenafil n = 30	p
**HbA1C %** Mean ±SD Range	5.58±0.69 4.5-6.5	5.41±0.55 4.5-6.35	0.276 I NS
**TSH (mIU/L)** Mean ±SD Range	3.06±1.53 0.5-5	2.51±1.15 0.78-4.78	0.118 I NS
**Prolactin (ng/ml)** Mean ±SD Range	15.30±6.14 2-25	13.03±6.80 2-25	0.181 I NS

n: number of cases, SD: standard deviation, I: independent samples t-test, NS: not significant

The ultrasound findings of the Nifedipine and Sildenafil groups are presented in Table 3. There were no significant variations in the average baseline pulsatility index between Nifedipine (2.02±0.52) and Sildenafil (2.03±0.49) (p=0.927). However, treatment with Sildenafil resulted in a more obvious reduction compared to treatment with Nifedipine, although the difference was not significant (p=0.152).

**Table 3:** Comparison of ultrasound characteristics between Nifedipine and Sildenafil groups

Characteristic	Nifedipine n = 30	Sildenafil n = 30	p
**Pulsatility index before** Mean ±SD Range	2.02±0.52 1.1-2.9	2.03±0.49 1.22-2.72	0.927 I NS
**Pulsatility index after** Mean ±SD Range	1.48±0.52 a 0.64-2.2	1.30±0.42 a 0.53-1.87	0.152 I NS
**Resistive index before** Mean ±SD Range	0.98±0.14 0.78-1.18	1.06±0.14 0.83-1.32	0.033 I *
**Resistive index after** Mean ±SD Range	0.70±0.07 a 0.59-0.82	0.62±0.06 a 0.52-0.73	< 0.001 I ***
**Endometrial thickness before** Mean ±SD Range	6.57±0.67 5.56-7.58	6.89±0.40 6.01-7.61	0.028 I *
**Endometrial thickness after** Mean ±SD Range	9.34±0.50 a 8.56-10.13	10.09±0.74 a 9.02-11.26	< 0.001 I ***

n: number of cases, SD: standard deviation, I: independent samples t-test, NS: not significant, *: significant at p≤0.05, ***: significant at p≤0.001, a: the results of paired t-test was that p<0.001

Regarding the resistive index, there was a significant variation in average baseline readings between enrolled categories, 0.98±0.14 *versus* 1.06±0.14, respectively (p=0.033), and treatment with Sildenafil resulted in a more obvious reduction than treatment with Nifedipine, and the difference was significant (p<0.001).

At baseline, the mean endometrial thickness of the Nifedipine group was significantly lower than that of the Sildenafil group, 6.57±0.67 mm *versus* 6.89±0.40 mm, respectively (p=0.028), but the difference was very small from a practical point of view (approximately 0.32 mm). Following treatment, the improvement in endometrial thickness attributable to Sildenafil was relatively better than that caused by Nifedipine, 10.09±0.74 mm *versus* 9.34±0.50 mm, respectively and the difference was significant (p<0.001).

The rates of adverse effects in the Nifedipine and Sildenafil groups are presented in Table 4. There was no significant variation in the rate of headache, flushing, vomiting, diarrhea, palpitation, and blurred vision between study groups (p>0.05).

**Table 4:** Rates of adverse effects in the Nifedipine and Sildenafil groups

Characteristic	Nifedipine n = 30	Sildenafil n = 30	p
**Headache** Positive, n (%) Negative, n (%)	6 (20.0 %) 24 (80.0 %)	8 (26.7 %) 22 (73.3 %)	0.542 C NS
**Flushing** Positive, n (%) Negative, n (%)	3 (10.0 %) 27 (90.0 %)	4 (13.3 %) 26 (86.7 %)	1.000 Y NS
**Vomiting and diarrhea** Positive, n (%) Negative, n (%)	1 (3.3 %) 29 (96.7 %)	2 (6.7 %) 28 (93.3 %)	1.000 Y NS
**Palpitation** Positive, n (%) Negative, n (%)	4 (13.3 %) 26 (86.7 %)	5 (16.7 %) 25 (83.3 %)	1.000 Y NS
**Blurred vision** Positive, n (%) Negative, n (%)	2 (6.7 %) 28 (93.3 %)	5 (16.7 %) 25 (83.3 %)	0.421 Y NS

n: number of cases, C: chi-square test; Y, Yates correction for continuity test, NS: not significant

## DISCUSSION

Recurrent miscarriage (RPL) in up to half of cases may have no known cause [3]. The only recognizable issue in a considerable number of women with RPL is aneuploidy of embryonic tissues, which may not have been evaluated before the referral to a specialized clinic. Because their examinations will almost always be normal, these individuals will be identified as having unexplained RPL. They are healthy individuals who have experienced unfortunate circumstances in their attempts to conceive. However, these women also have favorable chances of achieving future pregnancies without the need for medical or pharmaceutical interventions [20]. Well-designed meta-analyses and randomized controlled trials have demonstrated that intravenous immunoglobulin therapy, heparin and aspirin do not enhance outcomes of pregnancy in females with unexplained RPL, supporting this claim [21,22]. However, not all women with unexplained RPL have lost their pregnancies due to pure chance, and some may have other underlying issues that current investigation techniques fail to detect. These women, often younger and experiencing multiple miscarriages (4, 5, or more), pose the greatest challenge in terms of management [20].

Other than examining the embryo itself as a potential contributing factor to unexplained RPL, the capacity of the endometrium to distinguish between low-quality and high-quality embryos has received attention recently [23]. According to a preliminary investigation, preimplantation cytokines (such as prokineticin-1) were expressed at higher levels in women with RPL, making about 40% extremely fertile [24]. The researchers proposed that this extreme fertility prevents the natural process of preferring healthy embryos and permits the implantation of inferior embryos; therefore, pregnancy loss will happen. A following study looked at the migratory activity of endometrial stromal cells in response to low-quality and high-quality embryos to learn more about this [25]. However, other authors have suggested that endometrial blood flow and myometrial contractility may be blamed for the pathogenesis of unexplained recurrent pregnancy loss [17].

Therefore, in this study, two pharmacological agents were selected, one to enhance blood flow and the other to increase uterine blood flow and potentially reduce the risk of myometrial contractions, and compared their efficacy and safety in enhancing endometrial blood flow and thickness. The results of this study demonstrated that both agents were equally safe, as there were no significant differences in the rate of adverse effects. Additionally, both agents resulted in similar improvements in the pulsatility index. However, when comparing the resistive index and endometrial thickness, Sildenafil was more effective than Nifedipine. Sildenafil led to a greater reduction in the resistive index and a greater increase in endometrial thickness. These findings align with the results of Saleh *et al*., who showed in their randomized controlled clinical trial that Sildenafil was more efficient in improving endometrial thickness and uterine blood flow than Nifedipine [17]. This study supports these results, showing that both pharmacological agents were equally safe, but Sildenafil was more effective in reducing resistive index and increasing endometrial thickens. In line with these findings, previous studies by Huissoud *et al*. in 2004 [26] and Firouzabadi *et al*. in 2013 [27] demonstrated significant improvements in endometrial thickness and uterine blood flow with the use of Nifedipine and Sildenafil, respectively. These studies further support the results obtained in this study.

This study has several limitations, including small sample size and short duration, which may limit the statistical power and generalizability of our findings. Being a single-center study introduces potential bias and restricts the external validity. The significant variation in body mass index among participants could confound the results. Future studies should include larger sample sizes, longer follow-up periods, and multicenter designs to overcome these limitations and validate our findings. Controlling for confounding variables and considering diverse populations would improve reliability and generalizability. Further research should explore the long-term effects of Nifedipine and Sildenafil on pregnancy outcomes, optimize dosing regimens, and investigate combination therapies to enhance efficacy.

## CONCLUSION

In conclusion, both Nifedipine and Sildenafil demonstrated safety and efficacy in improving endometrial blood flow and thickness in women with recurrent pregnancy miscarriages. However, Sildenafil exhibited higher potency in achieving these outcomes.
